# Rheumatoid arthritis: pathological mechanisms and modern pharmacologic therapies

**DOI:** 10.1038/s41413-018-0016-9

**Published:** 2018-04-27

**Authors:** Qiang Guo, Yuxiang Wang, Dan Xu, Johannes Nossent, Nathan J. Pavlos, Jiake Xu

**Affiliations:** 1 Department of Spine Surgery, Xiangya Hospital, Central South University, No. 87, Xiangya Road, 410008 Changsha, China; 2 School of Biomedical Sciences, Faculty of Health and Medical Sciences, The University of Western Australia, Nedlands, Western Australia 6009 Australia; 3 Musculoskeletal Health Network, Department of Health WA, 189 Royal Street, East Perth, WA 6004 Australia; 4 School of Medicine, Faculty of Health and Medical Sciences, The University of Western Australia, Nedlands, Western Australia 6009 Australia

## Abstract

Rheumatoid arthritis (RA) is a chronic systemic autoimmune disease that primarily affects the lining of the synovial joints and is associated with progressive disability, premature death, and socioeconomic burdens. A better understanding of how the pathological mechanisms drive the deterioration of RA progress in individuals is urgently required in order to develop therapies that will effectively treat patients at each stage of the disease progress. Here we dissect the etiology and pathology at specific stages: (i) triggering, (ii) maturation, (iii) targeting, and (iv) fulminant stage, concomitant with hyperplastic synovium, cartilage damage, bone erosion, and systemic consequences. Modern pharmacologic therapies (including conventional, biological, and novel potential small molecule disease-modifying anti-rheumatic drugs) remain the mainstay of RA treatment and there has been significant progress toward achieving disease remission without joint deformity. Despite this, a significant proportion of RA patients do not effectively respond to the current therapies and thus new drugs are urgently required. This review discusses recent advances of our  understanding of RA pathogenesis, disease modifying drugs, and provides perspectives on next generation therapeutics for RA.

## Rheumatoid Arthritis: A preventable disease?

The preclinical stages of rheumatoid arthritis (RA) represent a golden window for the development of therapies which could someday prevent the onset of clinical disease. The autoimmune processes underpinning RA usually begin many years before symptoms such as joint pain and stiffness emerge. Recent studies have identified some of the key cellular players driving these processes and begun to unpick how genetic and environmental risk factors combine to trigger them; they also suggest the existence of several distinct subtypes of RA, which require further exploration. Jiake Xu at the University of Western Australia in Perth and colleagues review current treatment strategies for RA and how such insights could ultimately lead to the earlier diagnosis of RA - as well as providing new opportunities for drug treatment and prevention through behavioral changes in high-risk individuals.

## Introduction

Rheumatoid arthritis (RA) is a chronic systemic autoimmune disease that arises more frequently in females than males, being predominantly observed in the elderly. The prevalence rate reported in 2002 ranged from 0.5% to 1% of the population and had regional variation.^1^ RA primarily affects the lining of the synovial joints and can cause progressive disability, premature death, and socioeconomic burdens. The clinical manifestations of symmetrical joint involvement include arthralgia, swelling, redness, and even limiting the range of motion. Early diagnosis is considered as the key improvement index for the most desirable outcomes (i.e., reduced joint destruction, less radiologic progression, no functional disability, and disease modifying anti-rheumatic drugs (DMARD)-free remission) as well as cost-effectiveness as the first 12 weeks after early symptoms occur is regarded as the optimal therapeutic window.^2–4^ However, early diagnosis remains challenging as it relies heavily on the clinical information gathered from the patient’s history and physical examination supported by blood tests, and imaging analysis. The reasons for a delayed diagnosis vary markedly between countries with differing healthcare systems,^5^ while the reasons for a delay in initiating DMARD therapy in RA patients appear to be both patient- and physician-dependent. Noticeably, patient awareness of RA, the willingness of patients to seek medical advice, the time for the patients from symptom onset to receiving appropriate treatment, and the diagnostic capability of the physician all influence the treatment and outcome of RA. With poorly controlled or severe disease, there is risk that extra-articular manifestations such as keratitis, pulmonary granulomas (rheumatoid nodules), pericarditis/pleuritis, small vessel vasculitis, and other non-specific extra-articular symptoms will develop.

**Table 1:** Modern pharmacologic therapies for rheumatoid arthritis

**Classification**	**Name**	**Mechanism of action**	**Potential mechanisms**	**Side Effect**	**Reference**
Conventional synthetic DMARDs	Methotrexate	Analog of folic acid	Folate-dependent processes; Adenosine signaling; Methyl-donor production; Reactive oxygen species; Adhesion-molecule expression; Cytokine profiles Eicosanoids and MMPs.	Increased liver enzymes, pulmonary damage.	^83^
	Leflunomide/ Teriflunomide	Pyrimidine synthesis inhibitor	DHODH-dependent pathway; Leukocyte adhesion; Rapidly dividing cells; NF-kB; Kinases; Interleukins; TGF-β.	Hypertension, diarrhea and nausea, hepatotoxicity.	^153^
	Sulfasalazine	Anti-inflammatory and immunosuppression	Cyclooxygenase and PGE2; Leukotriene production and chemotaxis; Inflammatory cytokines (IL-1, IL-6, TNF-α); Adenosine signaling; NF-kB activation.	Gastrointestinal, central nervous system, and hematologic adverse effect.	^154^
	Chloroquine /Hydroxychloroquine	Immunomodulatory effects	Toll-like receptors; Lysosomotropic action; Monocyte-derived pro-inflammatory cytokines; Anti-inflammatory effects; Cellular immune reactions; T cell responses; Neutrophils; Cartilage metabolism and degradation.	Gastrointestinal tract, skin, central nervous system adverse effect and retinal toxicity.	^155^
Biological DMARDs Antibody-based therapies					
TNF-α targeted therapy	Infliximab	TNF-α inhibitor	Phagocytosis and pro-inflammatory cytokines; Chemoattractant; Adhesion molecules and chemokines; Treg cell function; Function of osteoclasts, leukocytes, endothelial and synovial fibroblasts.	Infection (pneumonia and atypical tuberculosis) injection-site reaction.	
	Adalimumab			Hypertension.	
	Etanercept			Severe /anaphylactoid transfusion reaction.	^156^
	Golimumab				
	Certolizumab pegol				
B-cell targeted therapy	Rituximab	B cell depleting	Fc receptor gamma-mediated antibody-dependent cytotoxicity and phagocytosis; Complement-mediated cell lysis; antigen presentation; B cell apoptosis; Depletion of CD4+ T cells.	Infection, hypertension, hypogammaglobulinemia, viral reactivation, vaccination responses.	
	Ofatumumab			Late-onset neutropenia.	
	Belimumab	Inhibitors of B cell function		Severe/anaphylactoid transfusion reaction.	^157^
	Atacicept				
	Tabalumab				
T-cell targeted therapy	Abatacept	CD28/CTLA4 system	Autoantigen recognition; Immune cell infiltrate; T cells activation.	Infection, malignancy.	^158^
	Belatacept	CD80/CD86			
Interleukin targeted therapy	Tocilizumab	IL-6 inhibition	Innate and the adaptive immune system perturbation; Acute-phase proteins.	Infections (most notably skin and soft tissue), increases in serum cholesterol, transient decreases in neutrophil count and abnormal liver function.	^159^
	Anakinra	IL-1 inhibition	Inflammatory responses; Matrixenzyme.	Injection site reactions, infections, neutropenia, malignancy.	
	Canakinumab				^160^
	Rilonacept				
	Secukinumab	IL-17 inhibition	Mitochondrial function; Autophagosome formation.	Infections, nasopharyngitis, candidiasis, neutropenia, safety data of mental health is limited.	^161^
	Ixekizumab				
Growth and differentiation factors	Denosumab	RANKL inhibitor	Maturation and activation of osteoclast.	Low Ca2+ and phosphate in the blood, muscle cramps, cellulitis, and numbness.	^162^
	Mavrilimumab	GM-CSF inhibitor	Activation, differentiation, and survival of macrophages, dendritic cells, and neutrophils; T helper 1/17 cell; modulation of pain pathways.	Safety file needs further research.	^143^
Small molecules					
JAK pathway	Tofacitinib	JAK1 and JAK3 inhibitor	T-cell activation, pro-inflammatory cytokine production, synovial inflammation, and structural joint damage.	Zoster infection (advice is to vaccinate beforehand) and other potential side-effects should be monitored carefully through further study.	
	Baricitinib	JAK1 and JAK2 inhibitor			^163, 164^
	Filgotinib	JAK1 inhibitor			
Future drug and target	Toll like receptors;^165^ Bruton’s tyrosine kinase;^151^ Phosphoinositide-3-kinase pathway;^166^ Transforming growth factor-beta;^167^ Neuropathways;^168^ Dendritic cell^169^				

While there is currently no cure for RA, the treatment strategy aims to expedite diagnosis and rapidly achieve a low disease activity state (LDAS). There are many composite scales measuring the disease activity such as the Disease Activity Score using 28 joints (DAS-28), Simplified Disease Activity Assessment Index (SDAI), and Clinical Disease Assessment Index (CDAI).^6^ To achieve full suppression of the activity of the disease (clinical remission), rheumatologists need to monitor disease activity continuously and accurately and to adjust the treatment regimen accordingly. Universally applied pharmacologic therapy with non-steroidal anti-inflammatory drugs (NSAIDs) and corticosteroids have proven effective in relieving stiffness and pain, but do not moderate disease progression. Over the last 20 years, the effectiveness of DMARDs has gained much attention as these can efficiently attenuate disease activity and substantially decrease and/or delay joint deformity.^7^ The therapy classification includes the traditional synthetic drugs, biological DMARDs, and novel potential small molecules. Historical DMARDs such as auranofin, minocycline, azathioprine, and cyclosporine are rarely implemented as modern therapies. Several biological DMARDs have recently emerged including TNF-inhibitor (Amjevita, Renflexis, Erelzi, Cyltezo, Imradl), anti-CD20 antibody (Truxima, Rixathon), IL-6 receptor antibody (Kevzara), RANKL antibody (Pralia), and JAK inhibitor (Olumiant). Despite the increasing number of new drugs and treatment regimes, complete long-term disease remission is not achieved for many patients and thus new therapeutic options are required. This review provides a contemporary appraisal of recent literature on the pathogenesis of RA and the potential of new pharmacological interventions for optimizing RA treatment regimes.

## Pathogenesis of RA

There are two major subtypes of RA according to the presence or absence of anti-citrullinated protein antibodies (ACPAs). Citrullination is catalyzed by the calcium-dependent enzyme peptidyl-arginine-deiminase (PAD), changing a positively charged arginine to a polar but neutral citrulline as the result of a post-translational modification. ACPAs can be detected in approximately 67% of RA patients and serve as a useful diagnostic reference for patients with early, undifferentiated arthritis and provide an indication of likely disease progression through to RA.^8,9^ The ACPA-positive subset of RA has a more aggressive clinical phenotype compared to ACPA-negative subset of RA.^10^ It is reported that ACPA-negative RA has different genetic association patterns^11^ and differential responses of immune cells to citrullinated antigens^12^ from those of ACPA-positive subset. In terms of treatment,^13–15^ less effective treatment response of methotrexate (MTX) or rituximab was observed in ACPA-negative subset. This suggests a requirement for future study on potential pathophysiology difference between these two subsets. For the purpose of this review, we will focus on the ACPA-positive subset of RA and divide the progression of RA process into several distinct stages. It is noteworthy to mention, however, that these stages may occur sequentially or simultaneously.

## Triggering stage

The appearance of ACPA is now widely used to diagnose and predict RA due to its high specificity (>97%) in clinical practice. ACPA occurs as a result of an abnormal antibody response to a range of citrullinated proteins, including fibrin, vimentin, fibronectin, Epstein-Barr Nuclear Antigen 1 (EBNA-1), α-enolase, type II collagen, and histones, all of which are distributed throughout the whole body. ACPA production has been associated with genetic and environmental factors. The strongest genetic risk factor associated with ACPA-positive RA is found in genes encoding HLA-DR, especially HLA-DR1 and HLA-DR4, also known as “shared epitopes” (SEs).^16^ It is thought that SE influences RA outcome via the production of ACPA and thus represents a primary risk factor for ACPA production.^17^ The protein tyrosine phosphatase non-receptor type 22 (PTPN22), which is a lymphoid specific protein tyrosine phosphatase, has also drawn much attention because of polymorphisms associated with ACPA-positive RA with the contribution of PTPN22 to ACPA-positive RA among various ethnicities.^18–20^ It may therefore act as a potent inhibitor of T cell activation and in turn affect in the ACPA production. Genetic variation of α1-antitrypsin has been found to be related to ACPA production in RA.^21^ However, whether the production is directly linked to α1-antitrypsin deficiency per se or results from altered autophagy induced by the mutant α1-antitrypsin Z requires further study. The increased response of type I interferon gene associated with Th2 cell induction and B cell proliferation correlates with ACPA production.^22^ Some researchers have recently compared the gene expression profiles between ACPA-positive RA and ACPA-negative RA patients.^11,23^ The critical solution to the puzzle is the association between the discovered genes and ACPA production. In addition, the risks of RA increase in individuals with a family history of RA. The risk of developing RA was three times higher in first-degree relatives of RA patients even though familial factors influence RA in men and women equally.^24–26^ It is also reflected in a twin study presenting recurrence risks at 9.5–13.1 in monozygotic co-twins and at 6.4–11.7 in dizygotic same sexed co-twins as opposed to a background population risk at only 0.37%.^27^ Another study of 12,590 twins reveals that environment, lifestyle, and stochastic factors may also play more important roles than genetics in ACPA production while genetic factors are more responsible for the progression from ACPA-positive individuals to arthritis.^28^

**Fig. 1:**
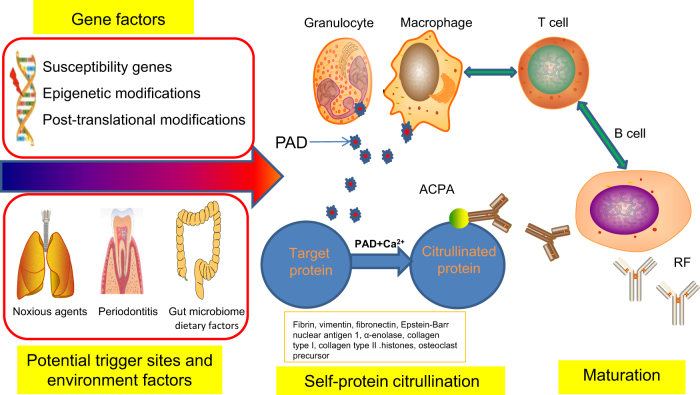
RA can be triggered in the potential trigger sites (lung, oral, gut, et al.) by the interaction between the genes and environmental factors, which is characterized by the onset of self-protein citrullination resulting in the production of autoantibodies against citrullinated peptides. Lung exposure to noxious agents, infectious agents (Porphyromonas gingivalis, Aggregatibacter actinomycetemcomitans, and Epstein-Barr virus), gut microbiome, and dietary factors may induce the self-protein citrullination and maturation of ACPA. Citrullination is catalyzed by the calcium-dependent enzyme PAD, changing a positively charged arginine to a polar but neutral citrulline as the result of a post-translational modification. In RA, PAD can be secreted by the granulocyte and macrophage. ACPA occurs as a result of an abnormal antibody response to a range of citrullinated proteins, including fibrin, vimentin, fibronectin, Epstein-Barr Nuclear Antigen 1, α-enolase, type II collagen, and histones, all of which are distributed throughout the whole body. Many citrullination neoantigens would activate MHC class II-dependent T cells that in turn would help B cells produce more ACPA. The stage is also called loss of tolerance. RA rheumatoid arthritis, PAD peptidyl-arginine-deiminase, ACPA anti-citrullinated  protein antibodies, RF rheumatoid factor.

The environment acts as a triggering factor for ACPA production in RA and the epigenetic regulation combines environment with genes. Gene–environment interaction influences the reactivity of autoantibodies to citrullinated antigens in RA.^29^ ACPAs can be detected long before the onset of the joint symptoms. This phenomenon suggests that the joints may not be the triggering spot for autoimmunity. Lung exposure to noxious agents, including smoke, silica dust, nanosized silica, or carbon-derived nanomaterials can trigger mucosal toll-like receptors (TLRs) that activate Ca^2+^-mediated PADs, but also antigen-presenting cells (APCs), such as classical dendritic cells (DCs) and B cells.^30–32^ The coatomer subunit α gene mutations could disrupt the endoplasmic reticulum (ER)–Golgi transport and cause hereditary autoimmune-mediated lung disease and arthritis, thereby providing a connection between the lung and the joint diseases.^33^ Moreover, smoking in the context of the HLA-DR SE gene may trigger RA-specific immune reactions to citrullinated proteins.^34^ DNA methylation mediates smoking and genotype interaction in ANPA-positive RA.^35^ There is ample evidence for three infectious agents regarded as autoimmunity triggers in RA, namely Porphyromonas gingivalis, Aggregatibacter actinomycetemcomitans (Aa), and Epstein-Barr virus (EBV). The periodontal space can also be a triggering site. In a clinic setting, 47% of the patients with RA showed evidence of previous Aa infection compared with 11% in the control group. The pathogen Aa can secret leukotoxin A and form pores in the neutrophil membranes that lead to neutrophil hyper citrullination, which results in the release of citrullinated autoantigens in the gums.^36^ P. gingivalis infection leads to citrullinated autoantigens and the ACPA production in two reported ways: one way is about PAD and arginine ginpains (Rgps) of P. gingivalis, which can cleave proteins at arginine residues and citrullinate proteins producing more neoantigens;^37^ another is about neutrophil extracellullar trap (NET) formation induced by the P. gingivalis during the process of NETosis. ACPAs induce NETosis and in turn NETosis provides citrullinated autoantigens.^38^ EBV can affect ACPA-producing B cells and impaired EBV control can be observed in RA.^39^ The intestinal tract is another mucosal organ implicated in the pathogenesis of RA because dysbiosis in RA patients can result from the abundance of certain rare bacterial lineages. It is well documented that gut microbiota may contribute to the pathogenesis of RA via multiple molecular mechanisms.^40,41^ Several studies have established the role of dietary factors in RA. The omega‑3 fatty acids might not only lower the risk of ACPA production but also prevent the onset of arthritis after detecting ACPAs.^42^ A healthier diet can also make a contribution to reducing the risk of ACPA-positive RA occurring at 55 years of age or younger.^43^ In addition, hormonal levels have been implicated in the pathology of RA,^44,45^ but the association with ACPA has not been firmly established. Alterations in gene expression regulation through both microRNAs and long non-coding RNAs have been proposed to contribute to the pathogenesis of RA. The contribution of other epigenetic modifications (e.g., sumoylation, histone methylation, histone acetylation, and deacetylation) and their functional role in RA currently remain unclear. Translation of above observations to effective treatment and exploring their interaction with the genome is challenging but would be meaningful. It is of significance to clarify the detailed knowledge of each risk factor in the triggering of RA so that tools can be developed to provide susceptibility scores and early diagnosis, as well as to identify new molecular targets for personalized medicine (Fig. 1).

## Maturation stage

This stage is initiated at the site of secondary lymphoid tissues or bone marrow. Epitope spreading refers to the development of immune responses to endogenous epitopes resulting from the release of self-antigens. The immune response to autoantigens may exist many years before disease onset and lay outside the joints. In this stage, epitope spreading and a gradually increased titer of ACPA can last several years before the onset of joint symptoms.^46^ Initial ACCP levels appear to be of great importance in predicting the interval time to disease onset.^9^ The production of ACPA reflects break of immunological tolerance. As a result, many citrullination neoantigens would activate MHC class II-dependent T cells that in turn would help B cells produce more ACPA. ACPA can induce pain, bone loss, and inflammation in RA.^47,48^ One study has identified that two RA-specific autoantigens N-acetylglucosamine-6-sulfatase (GNS) and filamin A (FLNA) correlate microbial immunity with autoimmune responses in the joint.^49^ What is more, it has been proposed that citrullination plays a unique role during osteoclast differentiation and ACPA-induced osteoclast activation which might explain important features of the gradual development of RA including why the joints are targeted. Other likely factors include biologic features of the targeted autoantigen, local microvascular, neurologic, and biomechanical factors, and microtrauma-related mechanisms may further contribute (Fig. 1).^50^

## Targeting stage

**Fig. 2:**
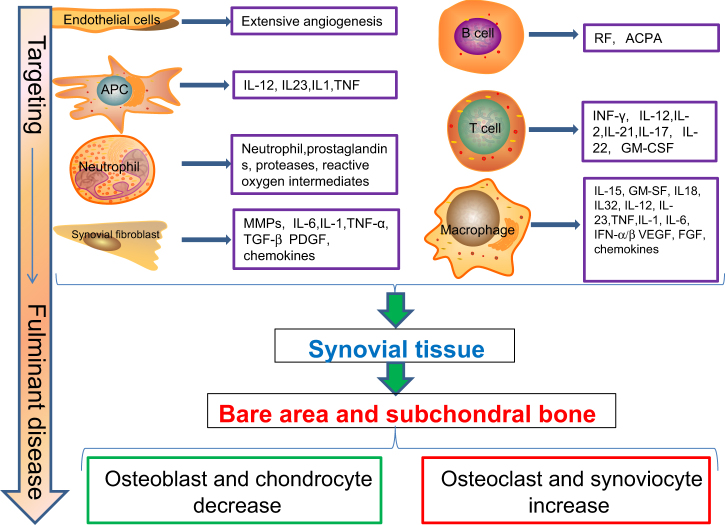
Many cells and their cytokines play critical roles in the development of RA. The synovial compartment is infiltrated by leukocytes and the synovial fluid is inundated with pro-inflammatory mediators that are produced to induce an inflammatory cascade, which is characterized by interactions of fibroblast-like synoviocytes with the cells of the innate immune system, including monocytes, macrophages, mast cells, dendritic cells, and so on, as well as cells of adaptive immune system such as T cells and B cells. Endothelial cells contribute to the extensive angiogenesis. The fulminant stage contains hyperplastic synovium, cartilage damage, bone erosion, and systemic consequence. Bone resorption virtually creates bone erosions, which are usually found at spots where the synovial membrane inserts into the periosteum, which is known as a bare area according to certain anatomical features. The destruction of the subchondral bone can eventually result in the degeneration of the articular cartilage as the result of a decrease in osteoblasts and an increase in osteoclasts and synoviocytes. IL interleukin, TNF tumor necrosis factor, MMP matrix metalloproteinase, TGF transforming growth factor, PDGF platelet-derived growth factor, IFN interferon, GM-CSF granulocyte–macrophage colony-stimulating factor, VEGF vascular endothelial growth factor, FGF fibroblast growth factor.

The involvement of RA in joints usually has a characteristic presentation with synovitis occurring in symmetrical small joints. Joint swelling is the external reflection of synovial membrane inflammation following immune activation. The normal synovial compartment is infiltrated by leukocytes and the synovial fluid is inundated with pro-inflammatory mediators that interact to produce an inflammatory cascade, which is characterized by the interactions of fibroblast-like synoviocytes (FLSs) with the cells of the innate immune system, including monocytes, macrophages, mast cells, DCs, and so on, as well as cells of adaptive immune system such as T lymphocytes (cell-mediated immunity) and B cells (humoral immunity). The two immune systems and their interactions are intimately involved in the development of ACPA-positive RA, which results in the failed resolution of inflammation (chronic synovitis). Monocytes/macrophages have been found to massively infiltrate synovial membranes^51^ and be central to the pathophysiology of inflammation. ACPA can enhance NF-kB activity and TNF-α production in monocyte/macrophages via binding to surface-expressed citrullinated Grp78.^52^ α-Enolase on the surfaces of monocytes and macrophages induces production of pro-inflammatory mediators.^53^ The imbalances between pro-inflammatory M1 macrophage and anti-inflammatory M2 macrophage must also be considered in the context of inflammatory RA.^54^ Indeed, a recent study reported that an imbalance in M1/M2 monocytes contributes to osteoclastogenesis in RA patients, especially in ACPA-positive RA.^55^ Further, the pro-inflammatory cytokine interleukin (IL)-17A in RA joint samples is localized primarily to mast cells based on one study^56^ and mast cells can be activated by ACPA and TLRs ligand.^57^ The accumulation of DCs in the articular cavity has also been reported.^58^ As an APC, especially myeloid DCs have been shown to induce T cell differentiation. A detailed understanding of how myeloid DCs function in RA may provide more effective RA treatment strategies. Other possible innate immune pathways comprise neutrophil NETosis, nature killer cell activation, etc. On the other hand, many researchers place the adaptive immune system at the center of RA disease pathogenesis. Most interest in the contribution of T cells has focused on their antigen-driven role and cytokine release of specific T cell subsets. CD4 effector T cells are major drivers of abnormal immunity in RA by sustaining chronic synovitis and supporting autoantibody production and a lack of reactive oxygen species could boost pro-inflammatory T cells, which shed light on the importance of energy metabolism in RA.^59^ As for B cells, the research focuses on their antigen presentation, antibody formation and release, and cytokine release into the milieu. Therefore, better understanding of the mechanisms of disordered innate immunity, including immune complex-mediated complement activation, adaptive immune responses against self-antigens, and abnormal cytokine networks may open up new avenues to restore immunologic homeostasis (Fig. 2).

## Fulminant stage

### Hyperplastic synovium

Synovium is characterized by a mixture of bone marrow-derived macrophages and specialized FLSs.^60^ Synovial cells maintain the steady state of the joint by secreting hyaluronic acid and lubricin for joint lubrication and function, as well as processing waste products. In RA, the dysfunction of FLS leads to hyperplastic synovium. The abnormal proliferation of FLS results from a loss of contact inhibition that plays a critical role in RA by producing inflammatory cytokines and proteinases, such as matrix metalloproteinases (MMPs) and tissue inhibitors of metalloproteinases (TIMPs) that perpetuate joint destruction. They create a microenvironment that allows for the survival of T cell and B cell and neutrophil accumulation.^61^ Another hypothesis regarding the cause of hyperplastic synovium is likely due to the resistance to apoptosis associated with distinctive pathways. Such pathways include abnormalities of tumor protein p53 function, which contributes to synovial lining expansion and joint destruction in RA;^62^ over expression of heat shock protein 70 and enhanced activation of heat shock factor 1 in RA synovial tissues that foster the survival of FLS.^63^ The pathogenetic mouse model synoviolin/Hrd1 triggers synovial cell outgrowth through its anti-apoptotic effects.^64^ It appears that synovial hyperplasia contains the proliferation of resident slow-cycling cells, such as mesenchymal stromal/stem cells and the infiltration of bone marrow-derived cells in lethally irradiated mice after bone marrow transplantation.^65^ Although animal models of RA have been useful, they do not always reliably replicate the human disease phenotype, even less the ACPA-positive RA.

### Cartilage damage

Cartilage acts as a key component of synovial joints, consisting of chondrocytes and a dense and highly organized extracellular matrix (ECM) synthesized by these chondrocytes and contains type II collagen and glycosaminoglycans (GAGs). The hyperplastic synovium causes major damage to the cartilage in RA through directed adhesion and invasion. Conversely, inflammatory signals, including those released from the ECM, can further stimulate FLS activity. The mediators of cartilage damage include MMPs, a disintegrin-like metalloprotease with thrombospondin type 1 motifs 4 and 5 and cathepsins. MMPs are synthesized by FLS and can promote disassembly of the type II collagen network causing biomechanical dysfunction. Membrane-type I MMP is envisaged to be the predominant proteinase that degrades the collagenous cartilage matrix.^66^ However, articular cartilage does not have enough regenerative potential by itself. Consequently, under the influence of synovial cytokines, particularly IL-1 and 17A, and reactive nitrogen intermediates, the cartilage is progressively deprived of chondrocytes that undergo apoptosis.^50^ This results in cartilage degradation demonstrable as joint-space narrowing on radiography. These observations may help explain why RA is a site-specific manifestation of a systemic autoimmune disease, in which early cartilage damage in the context of altered immune activation leads to a specific cellular activation of FLS within the articular joints.^67^ Nevertheless, a better understanding of the mechanisms underlying cartilage damage is required.

### Bone erosion

Bone loss is a pathological hallmark of RA and manifests as localized, periarticular and systemic bone loss. Bone loss is the result of the induction of osteoclasts and the suppression of osteoblasts. “Periarticular” bone loss most likely refers to cellular changes of the subchondral bone marrow, such as osteoclast differentiation and the formation of inflammatory infiltrates. It remains controversial whether inflammation or autoimmunity is the key driver for bone damage. Evidence for the traditional inflammatory theory is as follows: tumor necrosis factor alpha (TNF-α), IL-6, IL-1β, IL-17, and other inflammatory cytokines involved in RA could exert pro-osteoclastogenic effects and suppress bone formation in the appropriate environment via adequate signals, such as the receptor activator of nuclear factor kappa-B ligand (RANKL) and macrophage colony-stimulating factor (M-CSF).^68^ These promote the influx and differentiation of the monocytes into osteoclasts in the context of inflammation,^69^ while anti-inflammation therapies for RA arrest the progression of bone damage and vice versa.

The second possible pathway for bone loss in RA involves two mechanisms for autoimmunity that act as a trigger for structural bone damage. The first mechanism pertains to the formation of immune complex and Fc-receptor-mediated osteoclast differentiation. The second is the formation of anti-citrullinated vimentin antibodies against the most citrullinated protein, making osteoclasts the ideal antigenic targets for anti-citrullinated protein antibodies (ACPA). It is reported that ACPA binding to osteoclast precursors induces osteoclastogenesis, bone resorption, and bone loss.^70^ Bone resorption virtually creates a hole, which is usually found at spots where the synovial membrane inserts into the periosteum, which is known as a bare area according to certain anatomical features. Subchondral bone plays a vital role in maintaining the homeostasis of weight-bearing joints, and the destruction of the subchondral bone can eventually result in the degeneration of the articular cartilage. In the early stages of RA, bone marrow edema is a common finding at the spot of subchondral bone in humans,^71^ and aberrant transforming growth factor-β (TGF-β) in the subchondral bone is involved at the onset of RA joint destruction in animal models^72^ (Fig. 2).

### Systemic consequences

Multiple studies have documented an elevated risk of cardiovascular events in RA patients.^73^ The mechanisms responsible for this risk may be related to cytokines that increase endothelial activation and potentially make atheromatous plaques unstable. Patients with active untreated RA have reduced total cholesterol, low-density and high-density cholesterol.^74^ RA also influences the brain by causing fatigue and reduced cognitive function; the lungs by causing inflammatory and fibrotic disease; the exocrine glands by causing secondary Sjogren’s syndrome; the skeletal muscles by causing sarcopenia; and the bones by causing osteoporosis. Finally, RA patients may be at greater risk of cancer, especially hematologic and kidney cancers.^75^

## Modern RA pharmacologic therapies

The identification of a preclinical stage and a growing understanding of the natural history and mechanisms of RA development, alongside new potential therapeutic interventions, shapes the prospect that RA might be prevented in future.^76^ The current treatment principles for established RA involve symptomatic management and disease modification. A meta-analysis of 12 published studies confirmed that patients receiving delayed DMARDs therapy were at higher risk of developing radiographic joint space narrowing and bony erosions.^77^ In poorly controlled RA patients, bony erosions become evident on radiographs within 2 years of onset and these erosive changes are predictive of poorer functional outcome.^78^ In a patient with otherwise unexplained new onset polyarthritis, an urgent referral to a rheumatologist is thus mandatory to confirm an RA diagnosis and early initiation of a DMARDs-based treatment plan aiming for disease remission with prevention of deformity. Oral corticosteroids are potent and effective anti-inflammatory drugs that may contribute to disease modification.^79^ However, this needs to be weighed up against its well-known adverse effects. Symptomatic management remains important throughout the course of the disease and consists of everyday practical measures to deal with the primary symptoms of joint stiffness, such as pain and fatigue. Exercise is important to support joint flexibility and function, while abstaining from smoking is a universal advice to all RA patients given its impact on antibody formation. (Table 1.)

## Conventional Synthetic DMARDs (cs DMARDs)

### Methotrexate

MTX is a modified form of folate designed to have an increased binding affinity for dihydrofolate reductase (DHFR) compared with its parent molecule. MTX is the cornerstone in the treatment of RA either as a single agent or in combination with other DMARDs.^80^ In a recent meta-analysis, MTX showed a substantial clinical and statistically significant benefit compared to a placebo in the short-term treatment of people with RA, although its use was associated with a 16% discontinuation rate due to adverse side effects.^81^ Also, radiographic progression rates measured by an increase in erosion scores of more than 3 units were statistically significantly lower for patients in the MTX group.^82^ MTX has been proposed to participate in the process of folate antagonism, adenosine signaling, the blocking of methyl-donor production involved in reactive oxygen species, downregulation of the adhesion-molecule expression, modification of cytokine profiles, and the downregulation of eicosanoids and MMPs.^83^ Single nucleotide polymorphisms (SNP) analysis and genome-wide association studies (GWAS) have found some SNPs related to MTX responsiveness. For example, those located in the gamma-glutamyl hydrolase (GGH), 5-aminoimidazole-4-carboxamide (ATIC), and solute carrier family 19 member 1 (SLC19A1) genes.^84^ Nevertheless, the results from the studies are conflicting, and sufficiently large genomic studies are needed to further develop the understanding.

MTX for RA is administered as a low-dose (5–25 mg) weekly regimen with dosing conditional to the disease state and side effects. Oral MTX has a more variable uptake than subcutaneous administration, which also leads to fewer significant side effects. Subcutaneous MTX administration also demonstrated a greater bioavailability compared with oral MTX.^85^ MTX requires regular monitoring to optimize dosing and assess its immunosuppressive and hepatotoxic effects through frequent blood tests (monthly, initially). There are a few well-established drug interactions for MTX, including cotrimoxazole, which causes pancytopenia, combined with azathioprine or leflunomide, which causes liver and lung complications. NSAIDs can be safely used in conjunction with MTX for symptom control after over 30 years of routine use of the two agents. It is inconclusive that MTX enhances the risk of malignancy beyond the increased relative risk of neoplasia associated with RA per se.^81^ Despite this, the absolute risk is low. Adverse effects associated with the use of MTX additionally include the development of accelerated nodulosis, also known as MTX-induced accelerated nodulosis (MIAN), which occurs in (1–10)% of patients on MTX.^86^ However, most adverse effects can be reversed by supplementation with calcium or sodium folinate.^83^

### Leflunomide

Leflunomide reduces inflammation in the joints of RA patients by inhibiting dihydroorotate enzymes essential for producing DNA and RNA, particularly in activated proliferation lymphocytes. At higher doses, the active metabolite teriflunomide also inhibits tyrosine kinases responsible for early T-cell and B-cell signaling.^87^ Due to its different mechanism of action, Leflunomide is a valuable addition to the armamentarium of drug treatment for RA and is prescribed at a routine starting dose of 10 mg daily for the initial 3 days followed by 20 mg daily. Leflunomide has shown clinical, functional, and structural efficacy similar to MTX^88,89^ and has also been used effectively in combination with biological agents. Dose reduction to 10 mg daily should be considered if side effects occur, with the most common reported side effects being diarrhea, nausea, headache, rash, itching, loss of hair and body weight, hypertension, chest pain, palpitation, infection, and liver failure. It is thus important to monitor gastrointestinal symptoms, allergic reactions, alopecia, and liver function.^90,91^ There are a few well-documented drug interactions, including cholestyramine that impairs the absorption of Leflunomide, rifampin side effects caused by raising Leflunomide levels in the blood, and Leflunomide rarely increasing the anticoagulant effect of warfarin. Leflunomide is deleterious to developing fetuses and breastfeeding infants and therefore should be avoided during pregnancy and lactation.^92,93^

### Sulfasalazine (SSZ)

Owing to clinical trials, SSZ has been widely available as a therapeutic agent for RA because of its anti-inflammatory and antimicrobial activities. SSZ has significant efficacy in reducing active joint counts and slowing radiographic progression, which is comparable to the effects of Leflunomide.^94,95^ Its metabolites are sulfapyridine and 5-aminosalicylic acid (5-ASA). SSZ has the ability to increase the production of adenosine at the sites of inflammation; inhibit osteoclast formation via modulatory effects on the receptor activator of nuclear factor κβ (RANK), osteoprotegerin, and RANKL;^96^ inhibit TNF-α expression via the apoptosis of macrophages,^97^ and suppress B-cell function.^98^ Sulfapyridine may reduce IL-8 and monocyte chemotactic protein 1 (MCP-1) secretions in inflammatory cytokines.^99^ The common adverse effects of SSZ include gastrointestinal and central nervous system toxicity, rash, liver function abnormalities, leukopenia and agranulocytosis, megaloblastic anemia, oligospermia, and infertility. The way to minimize the side effects is the slow initiation of drug therapy and the serial monitoring of specific laboratory tests. There are no major drug interactions reported but patients should be cautioned about the risk and benefit ratio with pregnancy and breastfeeding.^100^

### Hydroxychloroquine

In RA, hydroxychloroquine is designed to interfere with the interaction between T helper cells and antigen-presenting macrophages that cause joint inflammation and decrease the production of pro-inflammatory cytokines, thus reducing the overall inflammatory response.^101^ Whereas, the classical explanation is that, while hydroxychloroquine impaired phago/lysosomal function, it also appears to work in a lysosome-independent manner by impacting on intracellular TLRs, particularly TLR9, by inhibiting the production of TNF, and by interfering with the processing of the conversion of the membrane-bound pro-TNF into soluble mature protein.^102^ Hydroxychloroquine has a gradual onset action of 2–6 months, demonstrating improvement of long-term functional outcome and retardation of radiographic damage.^103^ The common adverse effects are predominantly gastrointestinal, dermatological, and ophthalmologic. High dose and long duration of use of hydroxychloroquine act as risk factors for retinal toxicity which may progress even after cessation of hydroxychloroquine. Therefore, effective screening is important for early detection of retinal toxicity.^104^

## Biological DMARDs (bDMARDs)

**Fig. 3:**
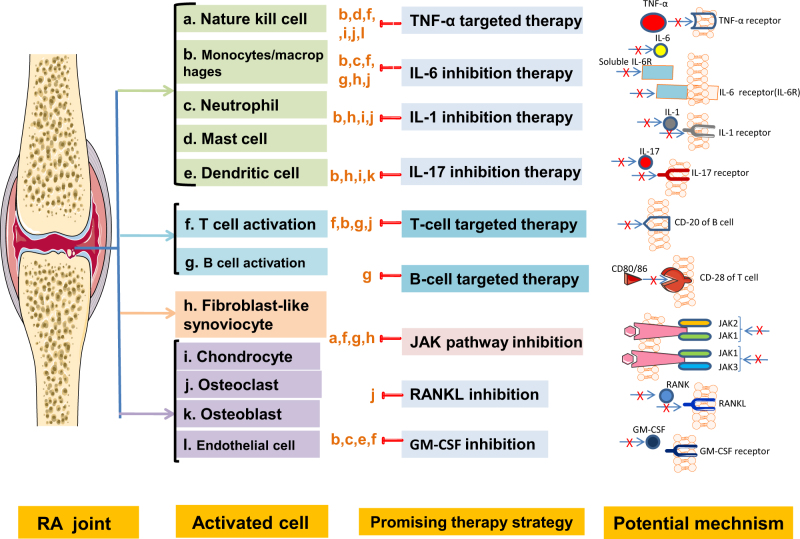
Cells and key receptors/pathways targeted by current therapy strategies. RANKL receptor activator of nuclear factor-ΚB ligand, JAK Janus kinase/signal transducers.

Although a somewhat vague definition, bDMARDs are a group of drugs that target specific molecules or molecular pathways involved in RA inflammatory processes. A number of bDMARDs have been shown to have clinical and radiological efficacy in the management of RA. TNF-α-inhibiting agents were the initial class of bDMARDs with newer agents targeting B lymphocyte antibodies CD-20, IL6, and CD28.^105^ (Fig. 3)

### TNF-α inhibitor (TNFi)

TNF-α triggers inflammatory responses and is produced by activated monocytes, macrophages, and T lymphocytes. TNF-α acts through TNF receptors 1 and 2, which have some species specificity and different affinity with TNF-α. Through the interaction of TNFα and its receptors, key signaling pathways can be activated, such as the NF-κB pathway, RANKL signaling, the extracellular signal-regulated kinase (ERK) signaling pathway, the tumor progression locus 2 (TPL2) pathway, and proapoptotic signaling. TNF-α has been proposed to mediate local bone destruction in the inflammatory musculoskeletal diseases due to the increased TNF-α levels in these diseases.^106^ TNF has been involved in the process of endothelial cell activation, the induction of metalloproteinases and adhesion molecules, angiogenesis, and the regulation of fibroblast/keratinocyte/enterocyte chondrocyte/osteoclast activation, as well as other inflammatory cytokines. Current evidence implies that TNF-α antagonists may ease arterial stiffness in RA.^107^ A substantial proportion of work-disabled patients with RA who start anti-TNF therapy regain work ability.^108^ Compared with patients with RA receiving sDMARD therapy, TNFi can decrease the risk of myocardial infarction.^109^ In the last 15 years, knowledge on the efficacy and toxicity of the TNFi has been published and was mainly gathered through regional or national registries created after these drugs reach the market. Based on the currently available literature, TNFi has, therefore, become the first choice of bDMARDs therapy in RA patients not responding to, or intolerant of, a conventional sDMARD treatment.^88^ Despite differences in biochemical and pharmacological properties of the five currently approved TNFi, there does not seem to be a clinically meaningful difference between them in terms of efficacy and safety. In a large cohort of RA patients, anti-TNF-α therapy does not increase the risk of serious bacterial infections compared with MTX therapy.^110^ This leaves the choice of TNFi mainly dependent on practicalities, such as dosing frequency or mode, or on wider economic considerations. In recent years, numerous biosimilar drugs have been developed, and some have already been approved. A biosimilar (bio-originator) refers a biologic medical product almost identical to an original product that is often produced by another company.^111^

Infliximab (IFX) was the first TNFi for RA treatment and consists of a recombinant chimeric monoclonal antibody composed of a human antibody backbone with a mouse idiotype. It can neutralize the biological activity of TNF-α by binding all forms of TNF-α. IFX is administered by intravenous infusion and in overall terms, IFX has an acceptable long-term safety profile.^112^ After the treatment with IFX in RA, a decrease of the adhesion molecule, IL-1, IL-6, IL-8, and MCP-1 was observed.^113^ Moreover, a reduced thickness of the synovial lining layer could be found.^114^ The IFX biosimilars include approved drugs in some countries, such as IFX-dyyb, SB2, CT-P13, BOW015, NI-071, PF-06438179/GP1111, STI-002, and ABP 710.^113^ IFX has adverse side effects, such as serious infections, the reactivation of hepatitis B or tuberculosis, and the risk of lymphoma and other cancers.

Adalimumab (Ada) is a fully humanized anti-TNF-α monoclonal antibody given by subcutaneous route fortnightly and has a less pronounced toxicity profile.^115^ Anti-Ada antibodies (AAA) are detected in more than half of the treated patients with RA. The AAA response is highly restricted and confined to the TNF-α binding region of Ada, thereby neutralizing its therapeutic efficacy and contributing to a loss of clinical efficacy.^116^ Ada is proven to be a potent antirheumatic agent to achieve remission and inhibit radiological progression. Furthermore, combination therapy with MTX is superior to monotherapy. The Ada biosimilars include drugs approved by some countries, such as ABP 501,^117^ Adfrar, and ZRC-3197.^113^ Ada has the adverse side effects, such as skin reactions, latent infections, and cardiac failure.

Etanercept is a recombinant protein composed of an immunoglobulin backbone and two naturally occurring soluble human 75-kDa TNF receptors. It is given by subcutaneous route twice weekly with toxicity profiles similar to IFX and Ada.^118^ Etanercept has shown sustained efficacy and function in rapidly decreasing radiographic progression in elderly and younger patients with RA.^24,119^ The number of patients achieving clinical remission with etanercept varies between 50% and 75% in the literature. Etanercept biosimilars include the approved drugs SB4 and GP2015.^113^

Golimumab is a human IgG1 kappa monoclonal antibody that binds to both the soluble and transmembrane bioactive forms of human TNF-α. It is administered once monthly by subcutaneous injection. While the short-term safety profile is reasonable with no differences in total adverse side effects, including serious infections, cancers, tuberculosis, or deaths. However, long-term surveillance studies are needed for further safety assessment.^120^ One-hundred milligrams of Golimumab showed numerically higher incidences of serious infections, demyelinating events, and lymphoma than 50 mg of Golimubab does.^121^ The Golimumab biosimilars include the BOW100 and ONS-3035, which are still in the preclinical phase.^113^

Certolizumab pegol is a human anti-TNF-α antibody Fab fragment that is chemically linked to polyethylene glycol and neutralizes membrane-associated and soluble TNF-α. It is administered every 2 weeks by subcutaneous injection and is well tolerated. Certolizumab pegol biosimilars include the PF-688, a drug still in preclinical phase testing.^113^ Significant side effects occur in 2% of people who take certolizumab pegol.^122^

Incidentally, TNFi (namely onercept and lenercept) failed clinical trials. However, TNF inhibitors have radically altered the approach to treat RA and have become an integral part of disease management. Medical professionals caring for patients should have the basic knowledge of its adverse side effects. Nevertheless, the inactivation of TNF signaling by rationally designed dominant-negative TNF variants needs further investigation.^123^

## **B-cell depletion and inhibition antibodies**

Rituximab is a genetically engineered chimeric monoclonal antibody that targets CD20-positive B lymphocytes from early pre-B-cells to later in the differentiation process, but it is absent in terminally differentiated plasma cells. The binding to CD20 enables rituximab to deplete subpopulations of B lymphocytes by way of cell-mediation, complement-dependent cytotoxicity, and the promotion of apoptosis and growth arrest. B lymphocytes may contribute to the initiation and maintenance of the inflammatory cascade by their action on antigen presentations and through the production of pro-inflammatory cytokines, including IL-1, -4, -6, -8, -10, and -12; TNF-α; vascular endothelial growth factor; MCP; macrophage migration inhibitory factor; and the autoantibodies rheumatoid factor (RF) and ACPA. It has been proposed that Rituximab has an effect on CD4+ cells, inducing substantial T-cell depletion in RA.^124^ Rituximab plus MTX demonstrated significant and sustained effects on reducing joint damage progression in RA patients who had a previously inadequate response to TNFi.^125^ The Rituximab biosimilars include the drugs BCD-020, Maball, and MabTas, which have been approved by some countries.^113^ The side effects reported include hypogammaglobulinemia, infection, late-onset neutropenia, and mucocutaneous reactions. Rituximab treatment has been linked with rare cases of progressive multifocal leukoencephalopathy (PML).

Belimumab is a monoclonal anti-B lymphocyte stimulator (BLyS) antibody. It binds to soluble human BLyS with high affinity and inhibits its biological activity. BLyS is elevated in the serum and synovial fluid of patients with RA and is associated with increased RF levels. The BLyS mechanism of action of is importance in the survival of B cells, and its inhibition can lead to the apoptosis of autoimmune B-cell clones.^126^ However, Belimumab was not effective in phase II clinical trials for RA. Other promising CD-20 targeting antibodies (obinutuzumab, ibritumomab, ocaratuzumab) need more clinical trials. The strategy of depth of depletion of B cell populations may not be the better way compared with the inhibition of B-cell modulatory cytokines.

## **T-cell targeted therapies**

Abatacept is a T-cell co-stimulation modulator and a fully human soluble fusion protein that consists of the extracellular domain of human CTLA-4, which is linked to the modified Fc part of human IgG1. T-cells infiltrate into the synovial joint and increase the level of pro-inflammatory cytokines such as interferon-γ and IL-17, causing synovial cartilage and bone destruction. Upon antigen recognition, T-cells require a costimulatory signal for full activation. Like the natural CTLA4 molecule, abatacept interferes with CD80/CD86 with higher avidity than CD28. Unlike other biologic drugs, it does not inhibit inflammatory proteins but blocks the communication between these cells by attaching to their surface. It is available in an infusible or injectable form and is administered to patients who have an inadequate response to one or more DMARDs. The data available on abatacept suggests the risk of serious infections when used together with the TNF-α blocker.^127^ Its side effects include headaches, common colds, sore throat, nausea, and infection. By contrast, targeting T cells using ciclosporin, anti-CD4 antibodies, anti-CD5 antibodies, or alemtuzumab have not yielded clinically robust responses in patients. The function of T cells and its subsets needs to be further reexamined.^128^ Other T-cell medications, such as ALX-0061, Sirukumab, Clazakizumab, Olokizumab, are still in the clinical trial phase.

## **IL-6 inhibition**

Tocilizumab (TCZ) is a humanized monoclonal antibody that targets the IL-6 receptor, which is found on cell surfaces and in circulation. IL-6 is produced by various cell types, including T cells, B cells, monocytes, fibroblasts, and endothelial and synovial cells. It has two receptors: mIL-6R (CD 126) and sIL-6R. In the pathology of RA, IL-6 can stimulate pannus formation through increased vascular endothelial growth factor expression and increase bone resorption as a result of osteoclastogenesis, as well as oxidative stress in leukocytes.^129,130^ TCZ is available in subcutaneous and intravenous formulations. Its immunogenicity risk is low.^131^ Decreases in neutrophil counts in patients taking TCZ do not appear to be associated with serious infections.^132^ Sirukumab, a human monoclonal antibody binding to the IL-6 with high affinity, also shows satisfied outcome with an expected safety profile in clinical phase 3 study.^133^ It provides another valuable chance to explore the effect of cytokine inhibition in RA rather than cytokine receptor inhibition. The most common adverse effects observed in clinical trials were upper respiratory tract infections, nasopharyngitis, headaches, and high blood pressure. The candidate IL-6 inhibitors currently undergoing clinical trials include sarilumab, ALX-0061, MEDI5117, clazakizumab, and olokizumab. Clinical trial data are promising and suggest that anti-IL-6 agents could be a promising therapy.^134,135^

## **IL-1 inhibition**

IL-1 is a cytokine that has the capability of immune and pro-inflammatory actions. There are two specific immunoglobulin-like membrane-bound IL-1 receptors, IL-1RI and IL-1RII. At the cell surface, IL-RII, in contrast to IL-1RI, does not transmit signals and acts instead as a decoy receptor that binds and inhibits IL-1. In serum, both IL-1 receptors can bind IL-1, thereby regulating the bioavailability of the cytokine.^136^

Anakinra (rHuIL-1ra) is a non-glycosylated recombinant form of the IL-1 receptor antagonist used as a once daily injectable. It is different from the native human protein by having an additional N-terminal methionine. It decreases the activity of IL-1α and IL-1β by binding to the IL-1 receptor. Its disadvantage includes the requirement of daily injections, and an itchy rash may be observed at the injection site. It can be used as a mono-therapeutic agent or in combination with DMARDs. However, anakinra should not be used in combination with anti-TNF agents. Its side effects include gastrointestinal tract reaction and allergy and infection of the upper respiratory tract; thus, it should be monitored carefully. Interestingly, RA patients receiving anakinra exhibited improved cardiac contractility even within 3 h of a single administration.^137^ Therefore, Anakinra should be considered for patients with severe or refractory pericardial disease and(or) heart failure.^138^ The benefits of IL-1 inhibition in this population are worth further exploration.

Other IL cytokines and their receptors have been studied as the potential target: IL-17 inhibitor (Secukinumab) was finished in a phase III study displaying improvement in patients with active RA who had an inadequate response to TNF inhibitors.^139^ However, IL-12/23 blockade, ustekinumab, did not see satisfying outcomes despite being combined with MTX in a randomized phase II study.^140^ The drugs targeting IL-7, 15, 18, 21, 32, and 33 are also in a clinical trial.

## **Osteoclast differentiation factor**

Denosumab (DMab) is a human monoclonal IgG2 antibody that inhibits bone resorption by binding and inhibiting the receptor activator of the NF-kB ligand (RANKL), an essential cytokine for osteoclastogenesis and bone resorption. Briefly, RANKL is an essential survival factor for DCs. RANKL-expressing Th17 cells mediate bone resorption. In addition, RANKL secreted by memory B cells promotes bone erosion in RA. Lastly, RANKL was known to induce immune tolerance by promoting the differentiation of Treg cells. It is conceivable that RANKL antagonists may influence immune regulation. The interplay of activated immune cells, synovial cell hyperplasia, and cytokine fosters an osteoclastogenic environment fueled by TNF-α and RANKL. Indeed, the presence of local and systemic bone loss in RA patients raised the possibility that the inhibition of RANKL may be an effective strategy to limit pathologic bone resorption.^141^ It has been proved that combining denosumab with DMARDs may be considered for RA patients with progressive bone erosions.^142^ Evidence from two phase II trials and one randomized observational trial indicate that DMab inhibits focal and systemic bone loss in RA. Phase III trials are required to discern the magnitude of the inhibitory effect on bone erosions and help to establish an optimal dose. The side effects include low Ca2+ and phosphate levels in the blood, muscle cramps, cellulitis, and numbness. Ultimately, DMab may prove to be a promising drug in the treatment of RA.^141^ Besides, the phase IIb study of a novel granulocyte–macrophage colony-stimulating factor (GM-CSF) receptor alpha monoclonal antibody, mavrilimumab, showed meaningful response by representing a novel mechanism.^143^

## Small-molecule DMARDs

Small-molecule DMARDs revolutionize RA treatment. Many cytokines use the Janus kinase (JAK) and signal transducer and activator of transcription (STAT) pathway to exert their effect in the pathology of RA, rendering them amenable to therapeutic blockade with Jakinibs which have proven effective for the treatment of RA.^144^ Jakinibs are being developed, and targeting STATs as well as other intracellular signaling pathways may be a future avenue for the treatment of RA, although substantial challenges remain.

Tofacitinib is the first of a new class of oral drugs to have synthetic small molecules that interfere with specific signal-transduction pathway and is the third class of DMARD (tsDMARDs) in RA treatment. It created the way to JAK inhibition in RA. Tofacitinib preferentially inhibits JAK-3 and -1 over JAK-2. With an oral bioavailability of 74% and mean elimination half-life of 3 h, tofacitinib is metabolized via cytochrome P450 3A4 (CYP3A4) with 30% renally excreted; 5 mg bd Tofacitinib has recently been approved by the FDA for moderate to severe RA refractory to DMARDs based on recent efficacy studies, with the onset benefits associated with the treatment occurring earlier.^145^ Common adverse side effects were related to infection, hematologic and hepatic disorders, and association of tofacitinib, with carcinogenicity and infections debatable.

Baricitinib is an orally administered molecular that inhibits JAK-1 and -2.  It has moderate activity on tyrosine kinase 2 (TYK2)and negligible activity on JAK-3 in both enzymatic and cellular assays. Baricitinib also proved effective in radiological progression. Peficitinib showed a 14 times higher selectivity for JAK-1/-3 over JAK-2. Filgotinib is a highly selective inhibitor of JAK-1 over JAK-2, JAK-3, and TYK2 in biochemical and cell assays. ABT-494 is also a JAK-1 selective Jakinib. Decernotinib that selectively inhibits JAK3 over the other JAK family members in both enzyme and cellular assays. The new Jakinibs with more restricted JAK isoform selectivity are now between phases 2 and 3 of clinical development. It is advised that jakinibs will require clinical and laboratory vigilance.^146^

## Future perspectives

With a better understanding of the pathophysiology of RA, new therapeutic approaches are emerging to provide precision medicine for individuals. However, the function and adverse side effects of these drugs will need to be carefully evaluated and used reasonably. Gene therapy means that treating RA by inserting a gene into a patient’s cells instead of using drugs.^147^ Targeting gene therapy in RA is a treatment strategy that is still in very early stages of development but could lead to new possibilities because of treating a disease at its root. The availability of Notch1 targeting siRNA delivery nanoparticles^148^ and TNF-α gene silencing using polymerized siRNA/Thiolated Glycol Chitosan Nanoparticles^149^ has been tested relatively successfully in an animal model. To prevent disease onset or relapses, smoking cessation or avoiding body exposure to environment risk factors is probably the easiest and most cost-effective method. Autoimmunity (tolerance break) develops years before the inflammatory phase of the disease, which can be considered as a golden period for preventing disease progression. Reestablishing immune tolerance and immunological homeostasis are ambitious goals in the way to overcome the disease. T cells and B cells can be targeted by specific drugs in the future to achieve seroconversion or delay the onset of joint destruction. Reduction of the function of APCs and modification of the pro-inflammatory properties of antibodies are being further developed.^150^ There is also a great interest in the novel approaches that have the possibility of becoming vital therapeutic targets, such as TLRs; Bruton’s tyrosine kinase; phosphoinositide-3-kinase pathway; TGF-β; neuro pathways, and DCs. Bruton’s tyrosine kinase is involved in various signaling pathways downstream of the pre-B-cell receptor and FcR, which is a promising therapeutic target for RA.^151^ The safety and tolerability of the intravenous infusions of expanded adipose-derived stem cells in refractory RA have been reported.^152^ (Table 1) In fact, new pathologic insight will support new avenues for therapeutic development.
